# Surgical Assessment and Post-Operative Complications Following Video-Assisted Thoracoscopic Surgery (VATS) of Horses with Severe Equine Pasture Asthma During Asthma Exacerbation and Remission

**DOI:** 10.3390/ani15152276

**Published:** 2025-08-04

**Authors:** Caitlin J. Wenzel, Cathleen A. Mochal-King, Alison L. Eddy, Jacquelyn E. Bowser, Robert W. Wills, W. Isaac Jumper, Andrew Claude, Cyprianna E. Swiderski

**Affiliations:** 1 Department of Pathobiology and Population Medicine, College of Veterinary Medicine, Mississippi State University, Starkville, MS 39762, USA; mochal@cvm.msstate.edu (C.A.M.-K.); aeddy@cvm.msstate.edu (A.L.E.); isaac.jumper@msstate.edu (W.I.J.); 2 Department of Equine Studies, Johnson & Wales University, Providence, RI 02903, USA; jbowser@jwu.edu; 3 Department of Clinical Sciences, Carlson College of Veterinary Medicine, Oregon State University, Corvallis, OR 97331, USA; claudea@oregonstate.edu; 4 Department of Companion Animal Medicine, College of Veterinary Medicine, Mississippi State University, Starkville, MS 39762, USA

**Keywords:** VATS, thoracoscopy, thoracotomy, equine asthma, pasture asthma, hyperinflation

## Abstract

The aim of this retrospective clinical study was to assess surgical duration and surgical and post-operative complications associated with Video-Assisted Thoracoscopic Surgery (VATS) and lung biopsy in horses with severe Equine Pasture Asthma (EPA) and paired control horses. Twelve horses (6 EPA-affected, 6 control) were sex, age and breed matched. Twenty-four thoracic surgeries were performed. Surgery of each matched pair (EPA-affected and healthy) was performed during asthma exacerbation (summer) and remission (winter). Surgical times were shorter with uncomplicated thoracoscopy (85 min) and significantly longer (*p* < 0.001) when intra-operative complications necessitated conversion to thoracotomy (156 min). The overall surgical time of EPA-affected horses during asthma exacerbation was significantly longer than control horses at any time point, predicted mean difference of 78 min (*p* < 0.05). When comparing EPA-affected horses to themselves during asthma exacerbation and remission, surgical times were significantly longer (*p* < 0.01) with a predicted mean difference of 98 min; this effect of seasonality did not occur amongst control horses. Intra-operative surgical complications (6/24) were evenly divided between EPA and control horses, however, only severe EPA horses in exacerbation were noted to have lung hyperinflation. Post-operative complications: fever, colic, hemothorax, pneumothorax, subcutaneous emphysema, surgical site infection, and/or laminitis occurred in 13/24 surgical procedures (54%). No fatalities resulted from these procedures.

## Simple Summary

Information on the post-operative complications and treatment of horses undergoing Video-Assisted Thoracoscopic Surgery (VATS) is limited. Postoperative outcome with long-term follow-up has not been described, nor has any study evaluated thoracic surgery in horses with severe Equine Pasture Asthma (EPA). This study evaluated the incidence of complications and surgical time in severe EPA horses with clinical disease, severe EPA horse in remission without clinical disease, and matched healthy, non-diseased horses undergoing thoracoscopy or conversion to thoracotomy for large pulmonary tissue biopsies using an endoscopic stapling device. Video-Assisted thoracoscopic surgery was performed on horses with EPA during asthma exacerbation and remission, and on their paired healthy control horse counterparts. This study found that during asthma exacerbation, which is triggered by grazing pasture in hot humid conditions (summer), surgical times in EPA horse were significantly longer. Horses with severe EPA may also experience lung hyperinflation which increases surgical difficulty. Complications associated with VATS and post-operatively are described.

## 1. Introduction

Endoscopic evaluation of the equine thorax has been practiced for over 40 years [1]. Standing thoracoscopy is documented in both healthy horses, horses with pulmonary disease, and cattle as an efficient and low risk tool for gross anatomical and pathological evaluation [2,3,4,5,6,7,8] of neoplastic disease, hyperinflation, diaphragmatic hernias [2,9,10], and diagnostic tissue sample collection [5,9,11,12]. For this reason, Video-Assisted Thoracis Surgery (VATS) with lung tissue biopsy is particularly suited for evaluating patients with diffuse lung disease, wherein focal tissue samples remain representative of the patient’s clinical disease state and are also large enough to support multiple diagnostic modalities.

The equine chronic respiratory disease referred to as severe Equine Pasture Asthma (EPA) has been previously referred to as heaves, chronic obstructive pulmonary disease, recurrent airway obstruction, summer pasture-associated obstructive pulmonary disease, and summer pasture recurrent airway obstruction [13,14,15,16]. Unlike the commonly referenced Equine Asthma (EA), that is associated with inhalation of moldy hay or dust aeroallergens in horses housed indoors, severe EPA occurs in horses grazing pasture during hot humid conditions (summer), predominantly in the Southeastern United States [14,15,16], or in the United Kingdom during the summer harvesting of crops [17]. Equine Pasture Asthma is a chronic, progressive, and debilitating disease that causes decreased quality of life, poor performance [18] and often progresses to severe airway obstruction and airway remodeling that can be fatal in horses not removed from the inciting environment [13].

Severe EPA is characterized by episodes of moderate to severe respiratory distress, bronchospasm, mucus accumulation, and airway inflammation. In contrast to horses with barn induced severe EA who respond to outside turnout, away from barn associated aeroallergens [13], horses with severe EPA are environmentally managed [13,15,16,19,20,21] with isolation from pasture-associated aeroallergens. In the severe EPA horse, this is done by housing the horse in a dry lot or stall. Horses with severe EPA exhibit TH-17 expression, marked neutrophilia in bronchoalveolar lavage fluid (BALF) [22,23], increased airway mucous production [22], persistent airway hyperresponsiveness [24,25], terminal bronchiolar remodeling [26], and evidence of decreased response to corticosteroid therapy as the disease progresses [24].

Video-assisted thoracoscopic lung biopsy has been performed in horses with barn dust associated EA in northern climates (Lansing, MI, USA; Montreal, QC, Canada). Exacerbation of barn dust EA was experimentally induced in these investigations. Exposure of EA horses to straw and hay allergens elicited respiratory compromise and neutrophilic airway inflammation, wherein the surgical procedure for lung tissue collection was performed using an endoscopic stapling device [5] or cautery devices [11,12].

Reports detailing post-operative complications and treatment of horses undergoing VATS are limited and postoperative outcomes with long-term follow-up have not been described. This study is the first to evaluate VATS and associated complications in horses with severe EPA. This study compares surgical time, the incidence of surgical complications, and post-operative complications in severe EPA horses during asthma exacerbation and remission and their matched healthy control horses undergoing VATS for the collection of large pulmonary wedge biopsies. We hypothesize that disease status will not have measurable effect on surgical time as demonstrated in the previous literature and that there will be minimal intra-operative and post-operative complications between horse groups.

## 2. Materials and Methods

### 2.1. Study Population and Inclusion Criteria

The horses and procedures described in this study reflect a previous research project supported by Agriculture and Food Research Initiative Competitive Grant no. 2015-67016-23172 from the National Institute of Food and Agriculture, United States Department of Agriculture. Animal use and procedures were approved by the Mississippi State University Animal Care and Use Committee and carried out under these regulations.

Twelve horses (6 severe EPA-affected and 6 non-diseased controls) were included in the study. Each cohort included three mares and three geldings. The breed distribution in each cohort was matched in 5/6 horses (Four Quarter Horse pairs, one stock breed pair, one Arabian pair, and one Quarter horse/Tennessee Walking Horse pair). The average age in each cohort was 19 ± 3 years (Range: 14–27 years old); horses were age-matched within 1 year in 5/6 pairs and within 3 years in 1/6 pairs. Horses were of standard size and weighed 473 ± 30 kg (Range: 420–534 kg).

### 2.2. Clinical Evaluation

Severe EPA was diagnosed based upon a well-documented history of three or more consecutive years of reversible airway obstruction associated with grazing pasture during summer in the Southeastern United States and associated neutrophilic airway inflammation on BALF cytology [22]. Healthy control horses were selected based upon absence of the previously described clinical diagnostic criteria for severe EPA for at least five years prior.

Based off this study’s research proposal, a physical examination, thoracic auscultation aided by rebreathing, complete blood count (CBC), serum chemistry, and cytologic examination of BALF were completed both at the beginning of the research project and preceding each surgery, to confirm appropriate cohort assignment [22] and rule out alternate or comorbid disease processes including pneumonia.

To identify periods of asthma exacerbation and remission, all horses were monitored daily, before 08:00, by trained staff, using the clinical score of respiratory effort (CSRE) [22] and incidences of additional clinical signs of asthma including wheezing, coughing, and paradoxical respiratory movement were also identified and recorded. Asthma exacerbation was diagnosed when CSRE ≥ 4.5/8 [22].

### 2.3. Experimental Design

Horses in this investigation had not been treated with medications used to treat clinical signs of asthma including corticosteroids, NSAIDS, and bronchodilators for at least a year prior to sampling. Severe clinical asthma exacerbations were managed using environmental modification if necessary. Horses with severe EPA and healthy control horses were co-housed with exposure to pasture-associated aeroallergens beginning 4 months prior to 10 June, which was previously identified as the first calendar day to reach 50% probability of EPA exacerbation [15]. Surgery during asthma exacerbation was timed to correspond to the patient’s first incidence of CSRE ≥ 4.5/8.

Between asthma exacerbation and asthma remission surgeries, EPA-affected horses experiencing asthma exacerbations were removed from inciting pasture to open-air stalls located approximately 500 feet from the inciting pasture. One horse failed to respond to environmental modification; due to continued exacerbation and severe airway hyperresponsiveness, the horse was moved into a climate-controlled barn where signs of asthma resolved.

Twenty-four VATS assisted lung biopsies were performed. Surgical site (i.e., right or left hemithorax) was random and evenly assigned to the diseased horses (3 right, 3 left) and replicated in the matched control horse. The opposite side was assigned for remission surgeries. The initial 12 surgical procedures were completed during seasonal summer disease exacerbation. The remaining 12 surgeries were completed during seasonal winter disease remission. Surgeries were randomly divided (13:11) between two ACVS diplomats (CM, AE) at Mississippi State University College of Veterinary Medicine. Following surgery, patients remained in the hospital’s intensive care unit for a minimum of 3 days, or until relevant complications resolved, horses were transferred to the progressive care unit until skin staple removal or complication resolution and then returned to pasture. The time from horses’ summer exacerbation surgery to winter remission surgery ranged from 112–293 days.

### 2.4. Pre-Operative Preparation

Twenty-four hours prior to surgery, each horse was bathed and returned to pasture. One hour prior to surgery, the horse was retrieved from pasture and placed in stocks in the veterinary hospital where they remained until ambulatory following recovery from sedation and the surgical procedure.

Patients were instrumented with a 14-gauge 13.3 cm polyurethane jugular catheter (Mila International, Hebron, KY, USA) and 21-guage Teflon (Terumo Medical Products, Somerset, NJ, USA) transfacial arterial catheter. Patients were administered flunixin meglumine (1.1 mg/kg, IV, Q12 h), gentamicin sulfate (6.6 mg/kg, IV, Q24 h), and either cefazolin (21 mg/kg, IV, Q8 h) or procaine penicillin G (22,000 IU/kg, IM, Q12 h) preoperatively.

### 2.5. Standing Restraint Anesthetic Monitoring

Standing sedation was overseen by a Board-Certified Veterinary Anesthesiologist (AC) and continuously monitored by a certified veterinary anesthesia technician. Horses were sedated with intravenous detomidine hydrochloride (0.01 mg/kg) and butorphanol tartrate (0.01 mg/kg) prior to being placed on a detomidine constant rate infusion (0.01 to 0.04 mg/kg/h), titrated to the desired effect. Horses were administered Lactated Ringers Solution at a 1–1.5 L/hour constant rate infusion. Oxygen insufflation (100%) was administered via nasal canula. Monitoring during standing sedation was initiated at time of the patient’s first sedation and concluded at the time of thoracic closure and the reestablishment of negative thoracic pressure. Monitoring parameters included heart rate (HR), rhythm, and cardiac function via base-apex ECG, respiratory rate (RR) and effort, mean arterial blood pressure (MAP) Q5 min, and blood oxygen saturation (SpO_2_) via pulse oximetry on the lip or tongue Q15 min.

### 2.6. Surgery

Following antiseptic preparation, a sterile ultrasound was performed on the hemithorax and its associated anatomy to confirm portal placement. Endoscopic portal locations were triangulated between the 11–16th intercostal spaces based off ultrasound findings. Sites were marked with a sterile skin staple prior to the administration of local anesthetic. Except for the predetermined portal placement via ultrasound, the VATS approach (induction of pneumothorax, initial biopsy approach with endoscopic stapler) was modeled after Lugo 2002 [5]. A pneumothorax was induced via teat canula through the first portal’s skin incision in the caudal dorsal lung field. After the induction of pneumothorax, a 30° 10 mm × 58 cm ridged laparoscope was inserted into the chest. An initial thoracoscopic exploratory was preformed first to observe for any intrathoracic abnormalities (i.e., free fluid, discoloration of the pulmonary tissue, abscess, fibrin or plural adhesions), and to confirm or alter the location of the previously determined thoracoscopic portals, based off the amount of lung tissue collapse observed endoscopically. A 12 mm trocar and cannula (Endopath 512 mm, Ethicon, Raritan, NJ, USA) was used in the most cranial ventral portal for the passage of the stapling device. A 45 mm linear stapling device (Endopath ETS-Flex 45, Ethicon, Raritan, NJ, USA) was used to biopsy the lung tissue through the 12 mm canula. Multiple staple deployments (2–5) were placed on the ventral border of the caudodorsal lung lobe to remove a wedge or elliptical shaped tissue sample averaging 3 × 5 × 1–2 cm in size. Once free from the parent lung, the tissue was moved into the 12 mm canula and withdrawn from the body.

Thoracoscopic procedures were converted to thoracotomy if the obtained tissue sample was too large to be removed via the 12 mm cannula or if the lung failed to collapse sufficiently after induction of pneumothorax due to pathologic hyperinflation. If the tissue sample was too large to be removed via cannula, the instrument portal was elongated approximately 2–3 cm and manual retractors were placed for atraumatic biopsy removal. In cases of lung hyperinflation, where the field of view was obstructed, the incision was lengthened to 15 cm and a Finochietto rib spreader was placed. The hyperinflated lung grossly was visualized, secured, and a 90 mm-tissue anastomosis stapler (TA90) was placed tangentially across the lung margin for biopsy acquisition.

After tissue transection and removal, the stapled margin of the lung was observed laparoscopically for hemorrhage or staple failure. Both thoracoscopy portals and sites converted to thoracotomy were closed in multiple layers (2–3) with 0 polydioxanone (PDS*II) (Ethicon, Raritan, NJ, USA) and skin staples. Before completion of deep layer tissue closure, a teat cannula was introduced through the most dorsal incision. Suction was used to reestablish negative thoracic pressure, and a preplaced suture was tightened as the teat canula was removed. Suction was utilized until the canula moved outward or the horse coughed. All incisions were covered with a sterile three-layer compression bandage. Horses remained confined to stocks until mentation and vital parameters (HR, RR, respiratory effort, temperature, SpO_2_) were within normal limits.

### 2.7. Post-Operative Monitoring

Each patient received gentamicin sulfate (Q24 h IV), procaine penicillin G (Q12 h IM) or cefazolin (Q8 h IV), and flunixin meglumine (Q12 h IV) for a minimum of 3 days. Patients that experienced complication(s) (fever (>312 K or 101.5 °F) [27,28], colic signs, hemothorax, post-operative pneumothorax, subcutaneous emphysema of the surgical site, or surgical site infection) were evaluated and treated individually by the project’s supervising internal medicine clinicians (CS, JB). Antibiotic therapy or analgesia protocol was amended dependent on the horse’s evaluation by the clinician. Patients remained under hospital care until skin staple removal (12–14 days) or until resolution of their post-operative complication(s). Severe EPA horses and controlled horses remained co-housed in the inciting pastures for 12 months following the last surgical procedure.

### 2.8. Statistical Analysis

The effect of thoracoscopy vs. thoracoscopy followed by incisional extension thoracotomy, severe EPA horses in exacerbation vs. control horses, severe EPA horses in remission vs. control horses, severe EPA horses in exacerbation vs. themselves in remission, sex, age, and breed on surgery time was assessed in separate linear mixed models using PROC MIXED. The effect of thoracoscopy, conversion to thoracotomy, severe EPA diseased, control non-diseased, patient’s 1st surgery, patient’s 2nd surgery, and side of hemithorax on each of the binary outcomes intra-operative respiratory event, colic, diarrhea, pneumothorax, hemothorax, fever, and surgery site infection was assessed in separate mixed model logistic regression models using PROC GLIMMIX. Similarly, these effects on the continuous outcomes Time, HR, RR, MAP, SpO_2_, and Days in ICU were assessed in separate linear mixed models. Random effects of horse identity within pair and pair were included in all mixed models. Using SAS for Windows v9.4, conditional residual plots were visually assessed to ensure the assumptions of normality and homoscedasticity were met by the linear mixed models. Random effects of horse identity within pair and pair were included in all mixed models. An alpha level of 0.05 was used to determine statistical significance.

Calculation of post hoc statistical power for surgical time was conducted using matched pair *t*-tests to assess the difference between two dependent means (i.e., surgical time in matched-pairs of horses) in G*Power 3.1.9.7 [29]. To inform power calculations, mean and standard deviation values for surgical time in all combinations of severe EPA horses vs. control horses in disease remission vs. severe EPA horses in disease exacerbation were used. Using matched-pair *t*-tests with a two-tailed approach, an alpha of 0.05, and the total sample size of 12 horses, the following statistical power was achieved for each comparison: severe EPA horses vs. control horses, disease exacerbation (power = 0.99); severe EPA horses vs. control horses, disease remission (power = 0.60); severe EPA horses, disease exacerbation vs. disease remission (power = 0.99), control horses, disease exacerbation vs. disease remission (power = 0.55).

Calculation of statistical power of complication (fever > 312 K (>101.5 °F), colic, hemothorax, pneumothorax, subcutaneous emphysema, surgical site infection, and laminitis) rate was conducted based on assessing the difference between two dependent proportions (i.e., complication rate in matched pairs of horses) using G*Power 3.1.9.7 [29]. When conducting within-pairs research (i.e., measurements are not independent), 0.25 may be used as a conservative estimate of discordant proportions [30]. Using the McNemar’s test of dependent proportions with a two-tailed approach and assuming an alpha of 0.05, the estimated odds ratio of 3.3 from and a total sample size of 12 horses, the computed power is 0.46.

## 3. Results

### 3.1. Sedation and Patient Monitoring

The ASA classification of each horse prior to surgery ranged from 1–3 (Mean: 1.9). ASA classification Mean for diseased horses during exacerbation and remission was 2.8 and 2.5, respectively. By contrast, ASA classification Mean for control horses was 1.1 during both surgical time points. Blood oxygen saturation the during the surgical procedure trended lower in horses with severe EPA (SpO_2_ Mean: 87%, Range: 77–97%) than in control horses (SpO_2_ Mean: 92%, Range: 78–100%), but this difference did not reach the threshold of statistical significance (*p* = 0.37). Monitoring parameters including RR, HR, tempreture, and MAP did not differ significantly between diseased horses and healthy control horses.

Five horses exhibited instances of respiratory distress during surgery as evidenced by increased respiratory rate, increased respiratory effort, and deteroiating SpO_2_. Of these five horses, four (3 severe EPA during disease exacerbation (summer), and one control horse during summer) responded to temporary closure of the thoroscopy portal(s) and evaculation of the hemithorax to reinflate the lung. Once the patient recovered, the surgical procedure was re-initiated and the biopsy sample was collected. One severly diseased EPA horse, in clinical remission, experienced an event of extreme respiratory distress after induction of pneumothorax (respiratory rate increased from 18 to 50 breaths per minute, marked increased abdominal effort, muscle fasciculations, and rapidly declining SpO_2_ to 82%, prior to intervention). Signs persisted in the face of lung reinflation. The horse’s portal was rapidly closed. The patient was quickly assisted to a recovery box, induced under general anesthesia, and received ventilator assisted breathing for 65 min; the horse regained spontanous breathing, normal respiratory character, and recovered uneventfully. Surgery was repeated on the same side, 89 days later; there were no complications.

### 3.2. Surgical Evaluations

All surgeries were initiated as VATS for lung biopsy; there were no abnormal intrathoracic findings appreciated on the initial thoracic exploratory other than the lung hyperinflation discussed below. Due to the required large tissue samples, the study design allowed a small extension of the portal incision (conversion to thoracotomy) for sample acquisition. Samples were retrieved via thoracoscopy in 11/24 cases (46%) and via thoracotomy in 13/24 cases (54%). Surgical time, summarized in Table 1, was significantly shorter in thoracoscopic surgeries (85 ± 62 min), than in thoroscopies converted to thoracotomy (157 ± 54 min, *p* < 0.001). Surgical time for EPA-affected horses during asthma exacerbation (190 ± 65 min) was significantly greater than control horses at any time (119 ± 41 min, *p* = 0.05). Additionally, surgical time for EPA-affected horses was significantly longer during asthma than during remission (75 ± 84 min, *p* < 0.01). Surgical times for EPA horses during remission (75 ± 84 min) did not differ significantly from control horses at any time point (119 ± 41 min, *p* > 0.05). Surgical time for control horses did not differ between time points.

**Figure 1:**
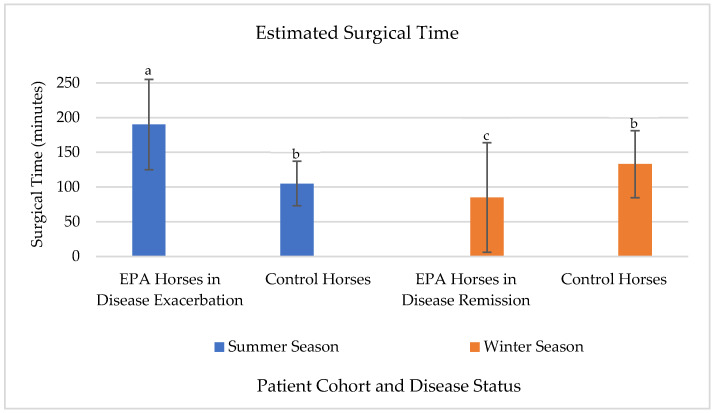
Seasonal effect of disease on surgical time using least square means estimate. Using a least square means, severe EPA horses in disease exacerbation during summer (a) were estimated to have a significantly longer surgical time when compared to control horses (b) at any time point. Severe EPA horses in disease exacerbation during summer (a) were also noted to have a significantly longer surgical time when compared to themselves during winter remission (c).

Using a least square means estimate (Figure 1), surgery performed on severe EPA-affected horses during summer asthma exacerbation was predicted to be 183 ± 23.8 min, and estimated on average to be 78 min ± 36.6 min (*p* = 0.05) longer than surgical times for control horses during any timepoint. Severe EPA affected horses during winter remission were predicted to have a surgical time of 98.2 ± 23.8 min, which was estimated to be 98 ± 20.7 min (*p* = 0.04) shorter than their previous surgery time during summer disease exacerbation. Age, breed, sex, lung laterality, and disease state had no effect on surgical time (*p*-value > 0.46).

Instances of surgical complications (Table 2) lung hyperinflation (3), insufficient insufficient tissue (2), friable tissue/tissue tearing (2), staple deployment failure (3), and loss of the wedge biopsy during delivery through the canula (3), occurred in 6/24 surgical cases (25%). Despite lung hyperinflation occurring only in EPA horses during disease exacerbation preventing thoracoscopic wedge biopsy, no significant relationship between surgical complication and disease state was appreciated.

### 3.3. Postoperative Evaluation

Thirteen of 24 horses (54%) experienced one or more of the following complications: fever > 312 K (>101.5 °F) (5), colic (9), hemothorax (3), pneumothorax (1), subcutaneous emphysema (6), surgical site infection (1), and mild laminitis (1). Complications were not significantly associated with any disease status. All horses with fever were noted to have an additional complication and were treated for the concurrent underlying condition (colitis, hemothorax, etc.).

Nine horses experienced signs of colic (ileus (3), pelvic flexure impaction (2), cecal impaction (2), colitis (2)). These horses were evenly distributed between severe EPA horses and controls. Patients demonstrating signs of colic were further diagnosed through physical examination (PE), nasogastric intubation, thoracic & abdominal ultrasound (US), and rectal palpation. Horses with colic signs related to ileus, pelvic flexure impaction, or cecal impaction were treated with one or more of the following: nasogastric intubation with gastric decompression, enteral fluid therapy via nasogastric tube (NGT), osmotic laxitive via NGT, mineral oil via NGT, intravenous fluid administration with electrolytes, and/or serial episodes of controled exercise as tollerated by their level of comfort post-operativly. Horses with evidence of colitis were further diagnosed though PE, CBC, thoracic and abdominal US, fecal smear, and subjective manure evaluation. These horses were treated with one or more of the following: polymyxin B sulfate (6000 IU/kg, IV, Q8 h), intravenous fluid administration with electrolytes, Chloramphenicol (50 mg/kg, PO, Q8 h) (one horse), di-tri-octahedral smectite (Bio Sponge, Platinum Performance) via NGT as needed, probiotic, and/or distal limb cryotherapy. Horse’s experiencing colic signs were already receiving flunixin meglumine as a part of their post-operative protocol. All patients responded favorably to conservative medical management and clinical signs of colic ceased within 24–48 h from the onset of clinical signs.

One severe EPA horse developed hemothorax following both surgeries. Hemothoracies were diagnosed via PE and thoracic US. These cases were managed as we have previously described [31], without drainage. Treatment included oxygen supplementation via nasal canula and antimicrobial therapy. The single incidence of pneumothorax diagnosed through PE (respiratory exacerbation) and thoracic US occurred in a control horse post thoracotomy. This was managed with supplemental oxygen via nasal canula flutter valve drainage, and intravenous 2% lidocaine constant rate infusion (0.05 mg/kg/min) for pain management.

Subcutaneous emphysema was recognized by smooth, normothermic, raised skin with crepitus near the surgical site and characterized as mild in all 6 cases. It was self-limiting. One surgical site infection at a thoracotomy incision was characterized by heat, mild swelling, and serous drainage. The distal 25% of the incision was opened and the site was flushed with dilute betadine solution followed by topical application of silver sulfadiazine until infection resolved. This same horse experienced a bout of presumed laminitis after an acute onset of unilateral hoof pain with responsiveness to a hoof tester over the toe. All patient complications were resolved by the time of suture removal, except the horse with laminitis that remained hospitalized for continued treatment.

While pain was not objectively evaluated, mentation and attitude were evaluated every hour while in the intensive care unit following their surgery. Subjective comfort level as assessed by attitude, full PE and surgical site evaluation was preformed regularly (Range: Q4 h–Q12 h). The patient medical records suggest that horses tolerated the post-operative period well. If discomfort or agitation was described in the daily progress notes, it was associated with a post-operative complication such as colic, hemothorax, or pneumothorax.

During the 12-month post-operative period in which horses were maintained in the research herd for additional studies, there were no new or relapses of post-operative complications. Severe EPA and control horses maintained their previously established respiratory characteristics with no increase in CSRE. Per the primary investigator of the original study, this remained true in subsequent years, until the horses were either euthanized due to age related conditions, disease progression, or lost to follow-up.

There were no fatalities associated with this study.

## 4. Discussion

### 4.1. Surgical Characteristics and Complications

This was the first study to examine complications of VATS and lung biopsy in horses with severe EPA. Thoracoscopic surgical time (85 ± 62 min) was notably longer compared to the previous report (28 ± 5 min) [5]. As the primary investigation supporting these surgical procedures required large wedge biopsies, additional tissue manipulation, staple deployment, and associated equipment complications may have necessitated more time. Moreover, the conversion to thoracotomy was not this study’s initial surgical goal and was only evoked as a contingency, as thoracic surgery is among the most painful surgical procedures described in human medicine. Pain from incisions, tissue damage and nerve damage is intense to extreme, may radiate from the chest to the back, and can progress to chronic and debilitating neuropathic pain that can last for up to a year [32]. As such, every effort to manipulate the biopsy sample into the canula, without causing tissue damage to the patient or biopsy, was attempted prior to the conversion to thoracotomy. The surgical conversion from thorascopy to thoracotomy resulted in significantly longer surgical times (157 ± 54 min), which was expected due to the additional patient preparations and change in surgical procedure required.

We demonstrated that surgery during clinical asthma exacerbation significantly increased surgical time in horses with severe EPA when compared both to controls and to themselves during disease remission. The within horse comparisons documented a significant (60%) reduction in surgical time when comparing severe EPA horses during asthma exacerbation to themselves during asthma remission. As asthma exacerbation in severe EPA occurs while grazing pasture during hot humid conditions, seasonality represents a mediating variable that significantly impacts disease exacerbation and associated surgical time. While the design of the original study required all surgeries of severe EPA horses during summer disease exacerbation and their controls to be performed first, and, all winter remission surgeries second, it is arguable that surgical skill and efficiency may have improved, thus decreasing surgery time. However, the seasonal reduction in surgical time was not identified in temporally paired surgical procedures of healthy control horses. We believe the effect of seasonality is further represented as 5/6 severe EPA horses necessitated conversion to thoracotomy during summer exacerbation surgeries, but all winter remission surgeries were completed as uncomplicated thoracoscopies, while control horses underwent mixed rates of thoracoscopy and thoracotomy throughout each season. Prior investigation of horses with experimentally induced barn dust EA, by inhalation of hay/straw aeroallergens in an enclosed barn, did not identify these differences in surgical times both within and between diseased and healthy groups [5,11,12]. Though many factors may account for this difference, we hypothesize that it may reflect a more severe asthmatic exacerbation for EPA horses in this investigation, whose asthma was naturally occurring rather than experimentally induced.

Surgical complications occurred in 6/24 surgical cases (3 EPA and 3 Controls); the rate of complication was not significantly associated with any experimental group due to equal cohort representation. Three of these six incidences included lung hyperinflation. This study recognizes lung hyperinflation the failure of the entirety of the lung field to collapse (approximated to maintain 50–70% of original size) during positive pressure instances (i.e., pneumothorax) as observed in non-diseased horse during both VATS and during postmortem evaluation [5,22]. Additionally, horses with lung hyperinflation exhibited rounded lung margins that remained physically in contact with the thoracic wall and diaphragm during thoracoscopic visualization. This insufficient lung collapse severely limited visualization during the VATS procedure, requiring conversation to thoracotomy for gross visualization in three diseased horses during asthma exacerbation as previously described. “Poor lung collapse” is only briefly mentioned in equine VATS procedures [5]. We determine the insufficient lung collapse in our study to be pathologic lung hyperinflation, a finding described in both barn dust and pasture-associated forms of severe equine asthma. This occurrence of hyperinflation occurred only disease exacerbation. As asthmatic horses undergo changes in the small airways, air becomes trapped in the terminal airways and alveoli. This decrease in airway function, both alveolar ventilation and functional lung volume, causes an increase in respiratory effort and volume in an attempt to restore normal ventilation, thus resulting a larger lung field and hyperinflation over time [13,22,26]. Despite the additional challenges of visualization, the average surgical time of these cases (190 min) did not differ significantly when compared to all cases converted to thoracotomy (157 min).

Surgical complications such as insufficient first tissue sample, friable tissue/tissue tearing, staple deployment failure, and loss of the wedge biopsy during delivery through the endoscopic canula expose the challenges associated with obtaining a large pulmonary biopsy via minimally invasive thoracoscopy. It is noteworthy to discuss that two of the horses with lung hyperinflation had additional complications of insufficient first biopsy sample, stapler failure, and loss of biopsy during thoracoscopic canula delivery. As this series of multiple complications did not reoccur in any other patients or during these patient’s remission surgeries, we hypothesize that each of these issues was either directly or indirectly associated with the patient’s lung hyperinflation as both visualization and tissue handling was challenging. Insufficient sample and staple failure are additionally attributed to the springform or elastic-like properties of the lung tissue caused by peripheral air trapping. Additionally, these complications may be also related to tissue thickness in the diseased lung as the anastomosing staples required a compressed tissue height of 2.0 mm for optimal tissue compression. While some horses experienced multiple complications, the complication incident rate (6/24) was not significantly associated with an increase in surgery time due to the distribution among cohorts.

Respiratory distress at the induction of pneumothorax was more common in diseased horses undergoing VATS during asthma exacerbation with 3 of the 4 instances occurring in this cohort. As horses have a fenestrated mediastinum [33], collapse of the contralateral lung field cannot be ruled out as a cause of respiratory distress following induction of pneumothorax. However, this would not explain the over-representation of respiratory distress at induction of pneumothorax in diseased horses and is more likely related to the EPA horse’s pathology resulting in poor pulmonary gas exchange and the horse’s inability to compensate during compromised ventilation as experienced during the induction of pneumothorax for VATS procedure.

We believe that due to the administration of 100% O_2_ via nasal canula, significant differences in SpO_2_ were not identified within or between groups. However, SpO_2_ in horses with severe EPA during both exacerbation and remission trended slightly lower than in control horses during any time point, a trend similar to a previous report with barn induced EA [5]. One severe EPA-affected horse had a pre-surgical baseline SpO_2_ of 84% associated with their surgery during summer asthma exacerbation. This horse’s thoracoscopic pulmonary wedge biopsy was successfully performed. Low SpO_2_ was attributed to the chronic lung pathology described in severe EPA horses [26], which is known to impair pulmonary gas exchange [5,12,34]. Additionally, ventilation/perfusion (V/Q) mismatching due to lung collapse can reduce arterial oxygen tension [3].

### 4.2. Post-Operative Complications

Post-operative complications including medical sequela were evaluated from the conclusion of surgery through an extended (12 months) post-operative observation period, which is a longer duration of observation than described for similar surgical procedures in previous literature of 48 h [5]. The post-operative all-cause complication rate was 13 per 24 surgical procedures (54%, *p* = 0.04). Eight of the 13 post-operative complications (62%) occurred in diseased horses, with 3 horses experiencing complications during both surgical events.

Nine horses experienced clinical signs of colic with 6/9 colic incidences due to gastrointestinal ileus or impaction. These nine horses were evenly distributed between both severe EPA and control horses and season. Ileus and delayed fecal output are well documented post-operative complications in horses undergoing standing sedation for procedures [35,36] and in humans undergoing thoracic surgery [37]. Possible mechanisms for post-operative ileus and impaction are multi factorial. Sedation with detomidine hydrochloride and butorphanol tartrate implicated in delaying gastrointestinal transit time in healthy horses [38,39]. Additionally, increases in sympathetic stimulation from noxious stimuli intraoperatively and postoperative pain may result in decreased gastrointestinal motility [36,40]. Due to the induction of pneumothorax and our population of severe EPA horses, respiratory components are considered, as both acute and chronic hypercapnia and hypoxemia are known to cause sympathoexcitation in humans with [41,42], further contributing to the incidence of post-operative ileus and impaction. While the current literature on VATS in horses does not describe this post-operative complication rate, our incidence of impactions 4/24 (17%) is nearly equal to the incidence of cecal impactions described in standing surgical procedures [36].

The three incidents of hemothorax noted in this investigation (12.5%) were comparable to the previously published findings (10%) for anastomosing staples [5]. One horse experienced two of the three hemothoraces. The pre-operative CBCs in both animals were normal; clotting factors were not evaluated prior to, nor at the time of surgery. The surgical fields were observed for hemorrhage after sample collection, but the biopsy site could not be fully observed following lung reinflation, making it impossible to rule out bleeding at that site. This is especially considered in one incidence, where the patient had lung hyperinflation and a limited field of view. Though a previous study cites iatrogenic diaphragm injury as possible cause of hemothorax [5], the horse that developed hemothorax after both surgeries was a Tennessee Walking Horse with noted narrow rib-interspaces [31]. We hypothesize that intercostal vessel injury and leakage, both well documented causes of hemothorax following percutaneous chest procedures, present a plausible etiology for the hemothorax observed following both procedures in this horse [43].

Our complication rate of pneumothorax (4.2%) was congruent to slightly improved compared to that of the previous report using anastomosing staples for lung biopsy (10%) [5]. Other study protocols have had increased rates of pneumothorax, upwards of 59–95% [10,11]. However, these reports used either a bipolar tissue sealing system or a ligating loop. In this project’s pilot study, a bipolar tissue sealing device was used in one horse, who soon died after. Due to the increased mortality associated with these sampling methods, anastomosing staples were utilized. Additionally, given the staple failure noted in 2/3 severe EPA horses with lung hyperinflation, we suspect that horses with severe EPA would be at a higher risk for life threatening complications, such as poor tissue sealing or tension pneumothorax in other sampling methods, as EPA lungs with hyperinflation displayed an elastic or spring back like character. Differentials for pneumothorax could include poor sealing of the lung tissue at sample collection due to poor tissue compression, failure of the staples after inflation of the chest, improper air evacuation of the thoracic cavity, and problems with incisional closure. Incisional leakage was considered the etiology of pneumothorax in this patient as covering the surgical site with an occlusive dressing proved instrumental in treatment. With the overall low incidence of pneumothorax, this surgical technique proved to be effective at mitigating tissue sealing issues, as previously documented [5], and provided adequate lung reinflation for post-operative patient ventilation.

Subcutaneous emphysema was documented in 6/24 (25%) of all cases. This occurrence rate was notably decreased from the reported 53–90% in cautery device biopsy sampling techniques [11,12], where skin portals were closed in a single layer. All horses who experienced subcuticular emphysema around the surgical site were within the control group. Two horses were noted to have repeated events of subcutaneous emphysema accounting for 4/6 of the recorded incidences. These two horses were documented as having a BCS of 6.5–7/9 which was at least 1 BCS higher than other control horses and all severe EPA horses. Increased incidence of subcutaneous emphysema could be associated with poor tissue closure due to tissue adiposity in horses with an increased BCS, or the lack of persistent lung pathology possibly resulting in more activity in the immediate post-operative period.

A single incidence of surgical site infection was recorded in association with the longest surgery time of 300 min. This procedure was completed in a diseased horse with lung hyperinflation during asthma exacerbation. Thoroascopic biopsy was attempted first, however, as the lung pathology grossly obscured the field of view, this surgery was converted to a 15 cm thoracotomy for sample collection via TA90 as previously described. As this horse had been documented to have pituitary pars intermedia dysfunction prior to entering the project, endocrine dysfunction, surgical duration, failure of the sterile field during conversion to thoracotomy, tissue trauma, and tissue hypoxia likely do to V/Q mismatch and other related EPA pathology were all likely precipitating factors for incisional infection in this patient [44]. Despite this patient’s endocrine dysfunction, this patient did not develop an incisional infection during asthma remission surgery remission surgery (225 min). Following remission surgery however, this patient developed our singular case of laminitis (in the left forelimb) four days post operatively. This mare’s pituitary dysfunction, chronic pre-study navicular disease, and post-operative colitis yield multiple etiologies regarding the inciting cause of laminitis.

Both VATS procedures and lung biopsies appeared well tolerated as there was minimal documentation of pain in the immediate post-operative period, a low incidence of potentially fatal surgical site associated complications (hemothorax, pneumothorax), no patient fatalities, and complications were not noted to reoccur or alter the phenotype of the horses in this study over a 12-month observational period.

## 5. Conclusions

This study evaluated surgical and post-operative complications of VATS in horses with severe EPA during asthma exacerbation and remission. A seasonal and disease effect on estimated surgical time was present, as EPA horses during exacerbation had significantly longer surgeries when compared disease remission and compared to healthy control horses’ surgeries. Lung hyperinflation in severe EPA horses posed a significant challenge and should be considered when performing this procedure in like populations. While the all-cause complication rate was significant at 54%, but no patients experienced any fatal complications. Albeit non-fatal, 25% of all patients experienced post-operative gastrointestinal ileus or impaction representing a notable, but common concern following standing VATS procedures. Rates of hemothorax and pneumothorax in this study were low and highlight the reliability of tissue anastomosing staples for lung biopsies. Surgical procedures were well tolerated in the immediate and 12-month post-operative period.

## Figures and Tables

**Table 1:** Surgical times and associated cohort, disease status/season.

Surgical Time (min)	Average Time	Standard Deviation	Minimum Time	Maximum Time	Number (Horses)
Thoracoscopy ^a^	85	62	35	225	(11)
EPA in Disease/Summer	85	-	-	-	(1)
EPA in Remission/Winter	84	-	-	-	(6)
Control in Summer	80	-	-	-	(2)
Control in Winter	92	-	-	-	(2)
Conversion to Thoracotomy ^b^	157	54	75	300	(13)
EPA in Disease/Summer	190	-	-	-	(5)
EPA in Remission/Winter	-	-	-	-	(0)
Control in Summer	118	-	-	-	(4)
Control in Winter	154	-	-	-	(4)
EPA surgeries	133	88	35	300	(12)
EPA in Disease/Summer ^c^	190	65	135	300	(6)
EPA in Remission/Winter ^e^	75	84	35	225	(6)
Control surgeries ^d^	119	41	50	180	(12)
Control in Summer	105	32	75	105	(6)
Control in Winter	124	52	50	124	(6)

Horses undergoing thoracoscopy (a) experienced a significantly (*p* < 0.001) shorter surgical time on average compared to horses undergoing thoracoscopy with conversion to thoracotomy (b) for sample delivery; no relationship between season or cohort was appreciated. Severe EPA horses in Disease Exacerbation during summer (c) were noted to have a significantly (*p* = 0.05) longer surgical time when compared to control horses (d). Severe EPA horses in Disease Exacerbation during summer (c) were noted to have a significantly (*p* = 0.0001) longer surgical time when compared to themselves during winter remission (e).

**Table 2:** Instances of surgical complications. Surgical complications represented in the table with an “🗙” represent a complication incidence of ≥1.

Instance	Cohort	Season	Lung Hyperinflation	Insufficient Tissue Sample	Friable Tissue/ Tissue Tearing	Staple Failure	Loss of Biopsy
1	Severe EPA	Summer	🗙		🗙		
2	Severe EPA	Summer	🗙	🗙		🗙	🗙
3	Severe EPA	Summer	🗙	🗙		🗙	🗙
4	Control	Summer				🗙	
5	Control	Winter					🗙
6	Control	Winter			🗙		

## Data Availability

The original contributions presented in the study are included in the article. Further inquiries can be directed to the corresponding author.

## Abbreviations

The following abbreviations are used in this manuscript:

BALF	Bronchoalveolar Lavage Fluid
CBC	Complete Blood Count
CSRE	Clinical Score of Respiratory Effort
EA	Equine Asthma
EPA	Equine Pasture Asthma
HR	Heart Rate
MAP	Mean Arterial Pressure
NGT	Nasogastric Tube
PE	Physical Examination
RR	Respiratory Rate
SpO_2_	Blood Oxygen Saturation
US	Ultrasound
VATS	Video-Assisted Thoracoscopic Surgery
